# Risk and protective factors for sudden infant death syndrome (SIDS) in low-resource communities in Kolkata India: a mixed methods exploratory study of semi-structured interviews and survey data

**DOI:** 10.3389/fped.2025.1652669

**Published:** 2025-11-20

**Authors:** Susan Samreth, Alexandra Leader, Jillian Kiely, Turja Chakrabarti, Rupa Kapoor

**Affiliations:** 1 Department of Pediatrics, Eastern Virginia Medical School at Old Dominion University, Norfolk, VA, United States; 2 Division of Pediatric Emergency Medicine, Department of Pediatrics, Northwestern University Feinberg School of Medicine, Chicago, IL, United States; 3 Department of Emergency Medicine, South Florida Baptist Hospital, Plant City, FL, United States; 4 President and Founder, Pratit International, Kolkata, India; 5 Department of Internal Medicine, Johns Hopkins Bayview Medical Center, Baltimore, MD, United States; 6 Department of Hematology and Medical Oncology, City of Hope National Medical Center, Duarte, CA, United States

**Keywords:** infant sleep, environmental factor, sleep environment, India, SUID (sudden unexpected infant death), SIDS (sudden infant death syndrome), infant care

## Abstract

Sudden Unexpected Infant Death (SUID), Sudden Infant Death Syndrome (SIDS) and infant sleep practices are widely documented and studied in high-resource countries. Knowledge of SUID/SIDS occurrence, risk factors and protective factors in low or middle-resource countries such as India is lacking. This was an exploratory study using a mixed methods (qualitative and quantitative) approach to better understand infant sleep practices and the various factors that may influence them amongst caregivers in 5 low-income communities in Kolkata, India. Twenty-eight and 22 caregivers of infants <12 months old were recruited using a convenience sampling approach to participate in semi-structured interviews (phase 1) and a survey (phase 2), respectively. This research was conducted in partnership with *Pratit International*, a locally based NGO dedicated to providing comprehensive health care to disenfranchised communities in Kolkata. Analysis of qualitative and quantitative data found that infant caregivers frequently described risk factors (e.g., bedsharing, soft sleep surface, infant sleep position, objects in sleep area, environmental smoke exposure, low rates of pacifier use) and protective factors (e.g., breastfeeding, routine immunization/prenatal care, low rates of caregiver substance use) that have been associated with SIDS in high resource communities. Qualitative data revealed that certain caregivers' reasons for infant sleep position were rooted in cultural beliefs. Prenatal and postnatal sleep education given by a healthcare professional were limited and awareness of safer sleep advice in the community was low. These study findings suggest the need to provide safer sleep education resources in a culturally appropriate manner and can help guide future research on potential interventions such as the baby box.

## Introduction

Sudden infant death syndrome (SIDS) is defined as the sudden unexpected death during sleep of a healthy infant under the age of one for which no underlying cause can be determined after thorough investigation including autopsy, clinical history, and circumstances surrounding the death (1). SIDS is a subset of sudden unexpected infant death (SUID), which is defined as a sudden and unexpected death which can be explained or unexplained (e.g., SIDS) and occurring during infancy (2). SUID is more readily described in low resource settings due to the difficulty in obtaining the resources (e.g., autopsy) to establish the diagnosis of SIDS.

In high resource countries such as the US, there are organizations advocating for safer sleep education and resources. The American Academy of Pediatrics (AAP) task force recommends supine position, firm sleep surface, noninclined sleep surface, breastfeeding/expressed breast milk, room sharing without bed sharing, avoidance of soft objects in bed (e.g., pillows, quilts, blankets), avoidance of overheating/covering infants head, avoidance of smoke, alcohol and illicit drug exposure during pregnancy and after birth, routine prenatal care and routine immunization and use of a pacifier which are all factors associated with decreased risk of SIDS in high resource countries (2). Although SIDS is one of the lowest reported causes of infant deaths globally, the prevalence of SIDS is still largely unknown in low resource countries (3). The reported number of infant deaths globally from SIDS is likely inaccurate due to underreporting, lack of awareness, or inability to diagnose SIDS.

It cannot be assumed that the risk factors for SIDS/SUID in high resource countries are generalizable. There is a paucity of literature describing sleep environment characteristics in low resource countries. The only large-scale, cross-cultural survey of infant and toddler sleep practices found that 64.7% of children in 12 (primarily Asian) countries/regions bed share and 87.5% room share, compared to 11.8% and 22%, respectively, in 5 (primarily Caucasian) countries (4).

In India, the infant mortality rate is 26 per 1,000 live births (5) and the prevalence of SIDS and SUID is largely unknown. In 2020, the third highest cause of infant mortality in India was “Ill-defined or cause unknown” (11.5%) (6). There are no studies describing the prevalence of SIDS/SUID or characterizing sleep practices of infants in India. Case studies, observational reports and cross-sectional studies in nearby countries such as Nepal, Sri Lanka and Saudi Arabia indicate that awareness of SIDS/SUID in local settings, even among healthcare providers, is low (7–9). Based on a published account from Nepal in 2005, infants who present to the emergency room deceased after hospital discharge did not undergo any further diagnostic investigations such as autopsy (7) In a study of pediatricians and pathologists regarding the rarity of SIDS in Sri Lanka, 87.5% had reported never encountering SIDS during their careers, and the majority of doctors believed the reason for this was due to infants being kept under close adult supervision during sleep as compared to infants in high resource countries (9). There is a need to characterize the awareness of SIDS/SUID and better elucidate potential risk and protective factors in the sleep environment in this setting.

The objective of this study was to establish qualitative and quantitative data to better understand infant sleep practices and the various factors (e.g., infant sleep education, maternal/caregiver practices, environmental exposures) that may influence them within several low resource, disenfranchised communities in Kolkata, India. The terms SIDS and SUID are often used interchangeably, but SIDS will be the term primarily used in this paper as it is most relevant to the risk factors and protective infant sleep practices described by study participants. While SIDS more accurately describes the sleep mortality of interest compared to SUID, the authors in this study recognize that the use of SIDS is not entirely accurate as the formal definition requires autopsy confirmation. Since there is no country-wide data or research dedicated to SIDS/SUID in India, this study will add to the literature and inform safer sleep education addressing the specific needs of this community.

## Materials and methods

### Setting

Caregivers from 5 different localities in Kolkata, India were included: New Metro, Dr. Ambedkar, Mathor Patty, Ramakrishna and Nivedita. The localities were home to between 150 and 1,100 people each (with a total estimated population of 3,000 people) at the time of the study. The majority were refugees from neighboring states of Bangladesh, Bihar, Uttar Pradesh, Jharkhand and Kashmir as well as the different districts of West Bengal. The primary spoken language was Hindi and Bengali and all of the localities were government-owned land. The estimated population of infants at the time of the study was 41 and the estimated population of children less than 5 years old was 252. All participants were part of the high need, low-income localities that partner with *Pratit International*, a humanitarian non-for-profit organization dedicated to providing comprehensive, sustainable health care in Kolkata.

The project was divided into two phases: 1) semi-structured interviews and 2) a formal survey. This project was reviewed and approved by the Human Subjects Committee of the Institutional Review Board (IRB # 17-07-EX-0179) at the Eastern Virginia Medical School (EVMS) and the Pratit International ethics board. The recruitment period for this study was from January 31st, 2019 to February 20th, 2019 for the semi-structured interviews and January 13th, 2020 to February 27th, 2020 for the survey. The phases of the project proceeded in a linear fashion with time between the phases to allow for survey revisions and internal validation, as well as to accommodate study logistics and travel. Participation was voluntary and no identifying information was collected. Informed consent was obtained from each participant.

### Phase 1: semi-structured interviews

This phase of the study used a qualitative exploratory design involving semi-structured interviews in a narrative approach. The authors adhered to the Standards for Reporting Qualitative Research (SRQR) checklist (10) to ensure complete and transparent reporting of the methods and findings.

In phase 1 of the study, a standardized interview script was developed with the goal of better understanding infant sleep practices and potential risk factors that may contribute to unexplained infant deaths and awareness of SIDS in the community. Interview questions were co-created with Pratit International community health workers to ensure cultural appropriateness, local relevance and readability. Topics included demographic information and questions regarding the infant's sleep environment, medical history and other risk factors for SIDS as well as caregiver knowledge of SIDS (Supplementary Image S1).

Semi-structured individual or group (4–6 participants) interviews were conducted by EVMS resident and attending physicians who went door-to-door in collaboration with Pratit International community health workers who served as interpreters and community liaisons. Participants were identified using a convenience sample approach and with the help of Pratit International community health workers who were familiar with the families residing in the localities and who had interacted with Pratit in the past. There was some overlap between caregivers who had completed the individual interview and the group interviews. Semi-structured individual interviews (a total of 28) relied on note taking of the interviewer. Group interviews (a total of 3) were audio recorded and subsequently transcribed (Supplementary Data Sheet S3).

While the sample included caregivers of young children, analyzed and reported data were restricted to caregivers of infants less than 1 year of age. Data were transcribed and coded independently by one researcher (SS) to generate themes. Frequencies were obtained on questions pertaining to infant sleep environment characteristics. A second researcher (RK) independently reviewed transcripts to confirm themes. Researchers jointly agreed on thematic saturation.

### Phase 2: survey development and implementation

In phase 2 of the study, a pre-piloting process was implemented by adapting a sleep survey designed for an international multi-site study on infant sleep practices conducted by EVMS and incorporating results from semi-structured interviews from phase 1 of this study. It should be noted that the interview questions on caregivers' SIDS awareness was not included in the 2020 survey as the study goal was focused on assessing the infant sleep environment and specific SIDS risk factors. The survey was translated into Bengali by community health workers certified to translate by Pratit International. The translated survey (Supplementary Image S2) underwent 3 iterations of respondent validation, or member checking, during which questions were either deleted, modified or added based on participants' and community health workers feedback on word choice, comprehensibility and cultural appropriateness. To enhance understanding between survey participants and interviewers, images and illustrations were used to describe the sleep surfaces. During the final iteration of the survey, each question was analyzed and discussed until there was consensus on the wording of the questions. The survey was piloted on 21 participants to assure clarity and feasibility. The final survey consisted of 50 questions.

In the final stage of phase 2, EVMS medical team researchers and Pratit International community health workers conducted the survey implementation phase of the project. Similar to study phase 1, a convenience sample of caregivers was recruited and written informed consent was obtained. Caregivers of infants less than 1 year of age were eligible to be surveyed.

Data was collected via door-to-door individual written surveys in four localities: Dr. Ambedkar, New Metro, Ramakrishna and Nivedita. Data were transcribed, entered into a password protected database system and personal identifiers were removed. Quantitative data, including demographic information, were summarized using frequencies.

## Results

### Study population

Demographics for the semi-structured interview and survey are contained in Table 1. Twenty-eight caregivers were interviewed in 2019; 22 caregivers were surveyed in 2020. Most interviews conducted in 2019 were among residents of the Nivedita locality whereas there was more diverse representation of localities in the 2020 survey.

**Table 1:** Demographic characteristics of participants.

Items	2019	2020
	Semi-Structured Interview	Survey
	n(%)	n(%)
Total number of participants	28	22
Infant's Gender		
Male	a	14 (64%)
Female	a	8 (36%)
Age of mother at time of delivery (years)		
(0–19)	a	5 (23%)
(20–29)	a	17 (77%)
(30–39)	a	0
(40–49)	a	0
(50+)	a	0
Route of delivery		
Vaginal (Natural) Delivery	a	12 (55%)
Planned C-section	a	4 (18%)
Emergency C-section	a	5 (23%)
Gestational Age at time of birth		
<7 months	a	0
7–8 months	a	1 (5%)
8–9 months	a	4 (18%)
≥9 months	a	16 (73%)
unsure	a	1 (5%)
Infant required NICU stay		
Yes	2 (7%)	3 (14%)
No	24 (86%)	19 (86%)
No answer	2 (7%)	0
Maternal education		
Primary (1st–4th standard)	a	6 (27%)
Secondary (5th–10th standard)	a	13 (59%)
Higher secondary (11th–12th standard)	a	2 (9%)
College/Graduate	a	1 (5%)
Other education (e.g., technical, trade school)	a	0
Current location of residence (Locality)		
Nivedita	13 (46%)	4 (18%)
Mathor Patty	5 (18%)	
Ramakrishna	3 (11%)	4 (18%)
Dr. Ambedkar	5 (18%)	8 (36%)
New Metro	4 (14%)	6 (27%)
Mother works outside home		
Yes	a	3 (14%)
No	a	19 (86%)
Father works outside home		
Yes	a	22 (100%)
No	a	0
Primary Caregiver		
Mother	a	19 (86%)
Father	a	0 (0%)
Grandparent	a	3 (13.6%)
Aunt/Uncle	a	2 (9%)
Friend or neighbor	a	0 (0%)
Sibling	a	0 (0%)
Number of other children being cared for by primary caregiver		
0 children	a	12 (55%)
1 child	a	7 (32%)
1–2 children	a	3 (14%)
>2 children	a	0

Data reported as counts (percentage) for all data above.

a This information was not collected in the 2019 semi-structured interview.

The majority of interviewees were non-working mothers under the age of 30 whose highest education was secondary school and who resided in the Dr. Ambedkar locality. Most infants were full-term males born via vaginal delivery who did not require a Neonatal Intensive Care Unit (NICU) stay and were the only child in the home.

### Phase 1: qualitative data

The following results were from analysis of semi-structured interviews from phase 1 of the study (Supplementary Data Sheet S3). These results informed key survey questions on infant sleep environment, environmental exposures and infant sleep education that were developed in phase 2 of this study.

#### Infant sleep environment

Caregivers identified the back and side positions for infant sleep. Surfaces identified included cot, mattress, floor, [plastic] mat, hammock and an enclosed soft bed. Blankets and pillows were objects described in the sleep environment. Caregivers typically prepared a sleep space after the baby was born.

#### Challenges at home

Participants highlighted several challenges when they came home with their baby including challenges with infant health, maternal health, the environment and finances. Infant health challenges included respiratory and digestive concerns, fever, and skin infections. Maternal health challenges included spontaneous miscarriages. Participants endorsed environmental concerns of rats, mosquitos and cockroaches and discussed using mosquito nets, electric mosquito repellants, solar lamps and mosquito coils to address these concerns. Although caregivers did not cite smoke exposure as a source of environmental concern, several participants endorsed the presence of cigarette smoke or cooking smoke (e.g., from wood fire, gas stove, kerosene oil) both inside and outside the home.

#### Infant sleep practices and advice

Participants cited a variety of reasons for placing their infant in a specific position to sleep. These reasons ranged from safety reasons (e.g., to prevent falls), medical reasons (e.g., for good head shape, to prevent vomiting, to prevent breathing problems) and cultural reasons (e.g., losing energy).

“If baby sleeps on back, shape of head will be good.”—Mother in Mathor Patty

“Sleep on back [to prevent] vomiting through the nose and prevent breathing problems.”—Mother in Ambedkar was told by her nurse and doctor

“If [baby] sleep on the side, baby's bones stick out and you have to massage it to get bone in the neck back in.”—Mother in Ambedkar

The above quote also suggests a possible cultural perception of “bones sticking out” if the baby were to sleep in the side position. Another caregiver cited fear of baby “losing their energy,” as a reason for putting their infant in the side position for sleep, as illustrated in the statement below:

“Lay baby on back and turn her to her side because if [she] lays on the back, the baby might get scared or lose their energy, so turn baby to side so baby wouldn't get scared.”—Mother at Nivedita, as told to her by her doctor

This reflects that some reasons for placing an infant in a specific position for sleep were rooted in cultural beliefs among caregivers and providers. One participant also endorsed negative perceptions of back sleeping, which was reportedly told to her by a state hospital personnel:

“Place baby on right side. If put on back, baby will die.”—Mother in Ambedkar

Sources of infant sleep advice based on semi-structured interviews were from family members (e.g., mother, mother-in-law), neighbors, providers and one participant cited a book provided by the hospital.

#### Awareness of infant death

Participants' understanding of SIDS was explored during the semi-structured interviews. Mothers shared their experiences of infant deaths during sleep, which appeared to be cases of suffocation.

“Baby had a cough/cold, went to sleep, mattress was [covering] mouth.”—Mother in Nivedita

“Heard of this happening where a baby had a breathing problem, blanket was over her face, baby was about 1 year [old].”—Mother in New Metro

One mother in particular shared that her second baby passed away at 1 month old during sleep:

“Her child that passed away breastfed. She doesn't want to remember it or share [further].”—Community Health Worker/interpreter assisting Mother in Ambedkar

The above statement reflects a feeling of repression or “not wanting to remember or share further,” and is one of many other emotions (e.g., sadness, shock, fear) caregivers described in regards to how the community reacted to an occurrence of infant death during sleep.

#### Baby box

Three of the semi-structured interview questions sought to gather feedback on caregivers' opinions of the baby box, a low-cost infant safe sleep space made from durable cardboard, equipped with a mattress and originally utilized in Finland, where it was associated with a significant reduction in SIDS/SUID (11). It has since been distributed in various global settings to promote safer sleep practices. However, there is a scarcity of reported needs assessments and cultural acceptability evaluations prior to distribution of baby boxes in various settings, as well a lack of reported impact/outcomes in the literature.

In this phase of the study, caregivers were shown a picture of the baby box on a tablet device and the baby box was described in detail. Participants were asked of their general impression. Participants generally had a positive impression of the baby box and frequently brought up the theme of safety.

“Baby is going to be safe, won't fall out, nothing can touch baby in there.”—Mother in Ambedkar

“[I] wouldn't worry about ants or rats, [I] would be worry free.”—Mother in Ambedkar

“Cats and other animals can't touch him.” –Mother in Ambedkar

“Baby falls from cot a lot, so this way baby won't fall out, will be safe. If she gets up and leaves, baby can fall, this way baby won't fall.”—Mother in Ambedkar

Participants did endorse some uncertainty with the baby box in regards to whether or not the box was compatible with mosquito nets and one participant expressed concern about suffocation:

“Worried about if you cover it [baby box], baby won't breathe”—*Mother in Ambedkar*

### Phase 2: quantitative data

Semi-structured interviews and survey data were used to collect quantitative data on the following topics: prenatal and well-baby checkups, sleep environment, infant care practices, environmental exposures and infant sleep education. Note that for a number of questions in the survey, respondents could select more than one answer (Supplementary Image S2).

Table 2 highlights that while the majority of mothers (21/22, 95%) went to all prenatal checkups, the majority of infants only went to the doctor when sick (13/22, 59%), although most mothers reported their children being up to date on vaccines (15/22, 68%).

**Table 2:** Descriptive statistics of prenatal and infant medical care practices stratified by 2019 semi-structured interview and 2020 survey.

Items	2019	2020
	Semi-structured interview	Survey
	n(%)	n(%)
Prenatal checkups		
Yes	25 (89%)	21 (95%)
No	1 (4%)	1 (5%)
Unsure/Did not answer	2 (7%)	0
Site of prenatal care		
Hospital	21 (75%)	
Did not answer	11 (25%)	
Routine checkups for baby		
Baby went to all visits	a	3 (14%)
Baby went to most visits	a	0
Only went to some visits	a	3 (14%)
None b/c baby <2 weeks old	a	3 (14%)
Only went to doctor when sick	a	13 (59%)
None	a	0
Routine vaccinations for baby		
Baby received all vaccines	a	15 (68%)
Baby received most vaccines	a	0
Baby received some vaccines	a	3 (14%)
None b/c baby <2 weeks old	a	3 (14%)
None	a	1 (5%)

All data reported as counts (percentage).

a This information was not collected in the 2019 semi-structured interview.

#### Infant sleep environment

Data on the infant sleep environment is shown in Table 3. Bedsharing and sleeping with objects were nearly ubiquitous. The most common reported items were a blanket (21/22, 95%), pillow (19/22, 86%) and sheets (19/22, 86%). 100% of interviewees endorsed that his or her infant slept with an object in the sleeping space and 82% (18/22) reported bedsharing.

**Table 3:** Descriptive statistics of infant sleep practices stratified by 2019 semi-structured interview and 2020 survey.

Items	2019	2020
	Semi-structured interview	Survey
	n(%)	n(%)
Most of the time, baby is placed in the following sleeping position^a^		
Side	5 (19%)	14 (64%)
Supine (Back)	16 (62%)	14 (64%)
Prone (Stomach)	2 (7%)	2 (9%)
More than one position	5 (19%)	
Leaning on object (e.g., pillow/blanket)	0	3 (14%)
No answer	2 (7%)	0
Reason for preferred sleeping position		
Advice from family/friends	4 (14%)	2 (9%)
Advice from healthcare provider	1 (4%)	2 (9%)
Other advice	0	0
Knowledge from personal experience	0	19 (86%)
Knowledge from electronic media (Internet, books, magazines)	0	6 (27%)
Baby's comfort	2 (7%)	1 (5%)
Safety concern	0	1 (5%)^b^
Not sure/No reason	13 (46%)	1 (5%)
Other	3 (11%)^b^	1 (5%)^c^
No answer	4 (14%)	
Bedding		During Day/During Night
Crib	0	1 (5%)/2 (9%)
Baby's bed (Portable Bed)	0	2 (9%)/1 (5%)
Hammock	2 (7%)	0/0
Bassinet	0	1 (5%)/0
Car seat	0	0/0
Playpen	0	1 (5%)/1 (5%)
Parent's bed (soft mattress)	18 (64%)	1 (5%)/4 (18%)
Parent's bed (hard mattress such as cot or *chowki*^d^)	9 (32%)	5 (23%)/11 (50%)
Rocker	0	9 (39%)/0
Mosquito net bed	0	1 (5%)/0
Floor	2 (7%)	1 (5%)/2 (9%)
Other	2 (7%)^e^	1 (5%)^f^/1 (5%)
Bedsharing		
Yes	22 (79%)	18 (82%)
No	2 (7%)	4 (18%)
No answer	4 (14%)	
Object in sleep area		
Pillow	7 (25%)	19 (86%)
Blanket	17 (61%)	21 (95%)
Soft mattress	1 (4%)	13 (59%)
Soft toys (e.g., stuffed animals)	0	3 (14%)
Hard toys (e.g., rattles, toy cars)	0	5 (23%)
Sheets	6 (2%)	19 (86%)
Clothes	0	11 (50%)
Diapers or Pampers	1 (4%)	5 (23%)
Bottles	0	7 (32%)
Other	0	1 (5%)^g^
None (baby does not sleep with any other objects)	3 (11%)	0

All data reported as counts (percentage).

a 2019 semi-structured interview did *not* utilize phrase “most of the time”.

b fear of vomiting.

c Headshape.

d *C*howki is a flat surface typically made of wood.

e Blanket, cloth.

f Quilt on floor.

g Mosquito net.

The most common sleeping area was the parent's mattress. Both soft (4/22, 18%) and hard mattresses (11/22, 50%) such as a cot or *chowki,* a flat surface typically made of wood, were reported. The most frequently reported infant sleeping position (note that respondents could select more than one answer) was on the side (14/22, 64%) and the back (14/22, 64%). The most commonly stated reason for the preferred infant sleeping position was “knowledge from personal experience (19/22, 86%),” followed by “electronic media (6/22, 27%).”

The 2020 survey also evaluated daytime vs. nighttime sleeping conditions. The most commonly reported sleep surface during daytime hours was a rocker (9/22, 41%). Night time sleep most commonly occurred on the parent's hard mattress/surface (11/22, 50%).

#### Infant care and maternal practices

As shown in Table 4, over 90% of caregiver mothers endorsed breastfeeding her infant (20/22, 91%) and no pacifier use (21/22, 95%). Reasons cited by mothers for discontinuation of breastfeeding include: infants remained hungry after breastfeeding (1/3, 33%), pain associated with breastfeeding (1/3, 33%), time required to breastfeed (1/3, 33%), and underproduction of milk (1/3, 33%).

**Table 4:** Descriptive statistics of infant care and maternal practices stratified by 2019 semi-structured interview and 2020 survey.

Items	2019	2020
	Semi-structured interview	Survey
	n(%)	n(%)
Baby ever received breast milk		
Yes	26 (93%)	20 (91%)
No	2 (7%)	2 (9%)
Baby currently drinking breast milk		
Yes	^a^	19 (86%)
No	^a^	3 (14%)
Duration that baby drank breast milk (for those not currently breastfeeding)		
Not currently but breastfed for <2 weeks	^a^	1 (100%)
Not currently but breastfed for 2 weeks-2 months	^a^	0
Not currently but breastfed for 2–6 months	^a^	0
Not currently but breastfed for 6–12 months	^a^	0
Reason that baby stopped receiving breast milk		
Difficulty latching or nursing	^a^	0 (0%)
Breast milk alone did not satisfy hunger	^a^	1 (33%)
Baby not gaining enough weight	^a^	0 (0%)
Mother's nipples were sore/cracked/bleeding	^a^	0 (0%)
Giving breast milk was too hard/painful or time consuming	^a^	1 (33%)
Mother was not producing enough milk/her milk dried up	^a^	1 (33%)
Too many household duties	^a^	0
It was the right time to stop breastfeeding	^a^	0
Mother became sick or had to stop for medical reason	^a^	0
Baby was jaundiced	^a^	0
Mother advised to stop giving breast milk	^a^	0
Other	^a^	0
Pacifier use		
Yes	^a^	1 (5%)
No	^a^	21 (95%)
Maternal Substance Use (smoking/drugs/alcohol) during or after pregnancy		
Yes	^a^	3 (14%)
No	^a^	19 (86%)
For those who answered yes, type of substance used		*During* pregnancy/Currently
Smoking	^a^	1 (33%)/0
Chewing tobacco	^a^	1 (33%)/2 (66%)
Alcohol	^a^	1 (33%)/1 (33%)
Drugs	^a^	1 (33%)/0
Other	^a^	0/0
For those who answered yes, using substances while simultaneously caring for baby		
Yes	NA	0
No	NA	2 (100%)

All data reported as counts (percentage).

a This information was not collected in the 2019 semi-structured interview.

The 2020 survey explored breastfeeding themes that surfaced in the 2019 semi-structured interviews, including questions related to maternal risk factors such as smoking, tobacco, alcohol or drug use. The majority of caregivers (19/22, 86%) denied any drug, alcohol or tobacco use during or after pregnancy, and all caregivers denied using any substances while simultaneously caring for the infant. When substances were reported, cigarettes (1/3, 33%), chewing tobacco (1/3, 33%) and alcohol (1/3, 33%) were the most common.

#### Environmental exposures

Table 5 highlights environmental exposures. The majority of caregivers (15/22, 68%) thought that their infant was exposed to tobacco or non-tobacco smoke in the home. While the majority of caregivers reported a smoker living in the home (15/22, 68%), less than half (7/15, 47%) perceived their infants were exposed to cigarette/cigar smoke inside the home. The most frequently reported source of smoke exposure was mosquito repellent/oils/incense burning (12/15, 80%), which correlates with mosquitoes being cited as the biggest perceived environmental threat. The second most frequently reported source of smoke exposure was smoke from cooking (11/15, 73%).

**Table 5:** Descriptive statistics of environmental exposures stratified by 2019 semi-structured interview and 2020 survey.

Items	2019	2020
	Semi-structured interview	Survey
	n(%)	n(%)
Does caregiver think baby gets exposed to any smoke		
Yes	^a^	15 (68%)
No	^a^	7 (32%)
Kind of smoke baby is exposed to		
Smoke from cigarettes/cigar exposure	10 (36%)	7 (47%)
Smoke from cooking (all sources)	^a^	11 (73%)
Smoke from wood burning for cooking	17 (61%)	5 (33%)
Smoke from coal burning for cooking	^a^	4 (27%)
Smoke from gas burning for cooking	^a^	2 (13%)
Smoke from kerosene burning for cooking	^a^	4 (26%)
Smoke from mosquito repellant coils/oil/incense burning	0	12 (80%)
Anybody in household smoke cigarettes or cigars		
Yes	10 (35%)	15 (68%)
No	14 (50%)	7 (32%)
No answer	4 (14%)	
Fuel source for cooking		
Wood/coal burning	1 (4%)	7 (31%)
Kerosene	0	6 (27%)
Gas	9 (32%)	12 (55%)
Cooking fire, unspecified	18 (64%)	0
Other		0
Perceived environmental threats		
Mosquitos or other flying insects	5 (18%)	20 (91%)
Scorpions other crawling insects	1 (4%)	17 (77%)
Rats/rodents	3 (11%)	17 (77%)
Snakes	0	7 (32%)
Flooding	0	2 (9%)
Other	0	5 (23%)^b^
None	1 (4%)	0
No answer	19 (68%)	0

All data reported as counts (percentage).

a This information was not collected in the 2019 semi-structured interview.

b Examples include: baby rolling off bed, poor weather, cats, dogs, fire.

#### Infant sleep education

Table 6 explores key data on reported education regarding infant sleep. The majority of caregivers (13/22, 59%) reported receiving education on infant sleep position, most often from a family member. In the clinical setting (e.g., from a healthcare professional), mothers most commonly received prenatal/postnatal education on breastfeeding (10/22, 45%) and the effect of medications on their baby (10/22, 45%), while recommended infant sleep positions (4/22, 18%) and smoke exposure (4/22, 18%) were among the least commonly discussed topics.

**Table 6:** Descriptive statistics of infant sleep education practices stratified by 2019 semi-structured interview and 2020 survey.

Items	2019	2020
	Semi-structured interview	Survey
	n(%)	n(%)
Someone (e.g., family member, nurse, doctor) discussed with caregiver how to lay baby down to sleep		
Yes	8 (29%)	13 (59%)
No	13 (46%)	9 (41%)
Unsure/Did not answer	4 (14%)	0
Who discussed how to lay baby down to sleep?		
OB/GYN doctor or nurse during the pregnancy checkups	0	0
Doctor nurse in the nursery	2 (7%)	6 (46%)
Baby's doctor	0	1 (8%)
Family Member	5 (18%)	8 (62%)
Friend/Neighbor	0	1 (8%)
Knowledge from personal experience	0	1 (8%)
Knowledge from electronic media (Internet/TV), books, magazines	0	0
Nobody	11 (39%)	0
Other	0	1 (8%)^a^
No answer	4 (14%)	
Position recommended by health care provider		
Side	^b^	3 (23%)
Supine (Back)	^b^	4 (30%)
Prone (Stomach)	^b^	0 (0%)
Leaning on object (e.g., pillow/blanket)	^b^	2 (15%)
No recommendation was given	^b^	5 (38%)
Position recommended by friend/family		
Side	1 (4%)	5 (38%)
Supine (Back)	4 (14%)	4 (31%)
Prone (Stomach)	0	0 (0%)
Leaning on object (e.g., pillow/blanket)	0	0 (0%)
No recommendation was given	0	5 (38%)
Mother received prenatal/postnatal education on any of following topics		
How to put baby to sleep	2 (7%)	4 (18%)
How smoking could affect baby	^b^	4 (18%)
Breastfeeding baby	^b^	10 (45%)
How drinking alcohol could affect baby	^b^	4 (18%)
How using narcotic drugs could affect baby	^b^	2 (9%)
How antibiotics or other medications could affect baby	^b^	10 (45%)
Unsure	^b^	4 (18%)

All data reported as counts (percentage).

a Social worker.

b This information was not collected in the 2019 semi-structured interview.

#### Baby box

After receiving the explanation of the baby box, 86% (19/22) of caregivers stated they would consider putting his or her baby in a baby box, while 14% (3/22) said they would not. Of those who would not use it, reasons included lack of comfort (1/3, 33%), lack of space (1/3, 33%) not wanting to put baby in a box (1/3, 33%), not wanting to put baby on the floor (1/3, 33%), and preference to have baby in the parents' bed (1/3,33%).

#### SIDS awareness

Eighteen percent (5/28) of caregivers had heard of an infant dying during sleep. Of these mothers, the anecdotal cases were described in infants ranging from 4 days to 6 months of age. Three incidents of infant deaths with identifiable causes were also reported to occur: 1) Infant death during breastfeeding, 2) Infant death due to breathing issues, and 3) infant death due to trauma after a fall into a ditch. After excluding these 3 cases, 2 examples of sudden infant death syndrome were reported.

## Discussion

This was an exploratory mixed-methods study given the scarcity of data on infant sleep practices and SIDS in low- and middle-income countries (LMIC), and particularly among marginalized communities. This study revealed that there were many potential risk factors for SIDS reported in the home environment and infant sleep practices of families in the Kolkata, India communities surveyed. Major categories of SIDS risk factors that were present included: bedsharing, soft sleep surface, infant sleep position, objects in sleep area, environmental smoke exposure, low rates of pacifier use and lack of safer sleep education resources.

Over 80% of caregivers in this study reported bedsharing, which is higher than other studies on infant sleep practices in this region (4). While in the U.S. the AAP advises avoidance of bedsharing, the authors in this study recognize that safer sleep guidance does vary by country. For example in the UK, Norway and Australia, experts do not routinely advise against *all* bedsharing but they do advise against bedsharing in the presence of specific risks such as soft bedding (e.g., sofas), the presence of any smoking, drug or alcohol use or the presence of infant vulnerability (e.g., an infant with history of prematurity or small for gestational age) (12–14).

Over 2/3 of infants in this study were exposed to non-tobacco smoke (e.g., from a cooking fire). While there is insufficient evidence regarding the risk of SIDS from non-tobacco smoke, guidelines from high resource countries recommends eliminating *all* sources of smoke exposure in environments where infants reside (2). The low pacifier use in this community was much lower compared to other studies in nearby countries such as Saudi Arabia, where a cross sectional study of 200 participants reported a pacifier use of 58% (15). One possible reason for this finding is research by the Indian Maternal and Child Health Association which reported ill effects of pacifier use in children (16).

Lack of safer sleep education resources may be attributed to lack of educational resources in clinical settings, lack of knowledge or awareness of SIDS among health care professionals, and lack of attendance to routine infant checkups and a general propensity to only take the baby to the clinic or doctor when the child is sick. The findings of the perceived environmental threats in this study were also informative, as they lend important considerations to providing infant sleep education that takes into account the large concern for mosquitos/flying insects, scorpions and rats/rodents and the potential use of mosquito nets in the sleep environment.

Protective factors for SIDS reported by participants include high rates of breastfeeding, high rates of routine immunization and prenatal care, and low rates of caregiver substance use prenatally and while caring for the infant. Interestingly, this study found that while few infants went to routine pediatric checkups, the majority of infants were up to date on their vaccines. It was unknown *where* the majority of these infants were getting vaccinated (e.g., in government-run or NGO-sponsored vaccine clinics or in the private setting). Such information would be useful in targeting settings for infant sleep educational campaigns, especially since routine immunizations are protective against SIDS in high resource settings (2).

Caregiver's awareness of SIDS or infants passing away unexpectedly during sleep during the first year of life was overall higher than expected. Since the prevalence of SIDS in India and are relatively unknown, it was hypothesized that there would be little to no awareness of SIDS. Approximately 7% of caregivers in this study reported awareness of a case of SIDS. This is lower than other studies in the region, such as a study in Saudi Arabia which reported a 26.6% awareness level of SIDS among mothers (8) and a study in Sri Lanka which reported 12.5% of surveying physicians had diagnosed SIDS during their professional career (9).

The qualitative phase of the study revealed that the topic of SIDS was a difficult one to bring up, as some participants seemed to be taken aback or surprised by the topic or did not want to discuss the topic at all. Caregivers who had shared their personal experiences of infant deaths during sleep were frequently noted to be emotional and seemed to still carry the grief from the event, demonstrating the potential impact of SIDS in this community.

Semi-structured group interviews revealed that mothers had both positive (e.g., good head shape, prevent vomiting/breathing problems) and negative (e.g., death, infant fear or losing energy) motivating factors for placing their infant in the back position to sleep. This suggests the need for factoring culturally tailored advice when it comes to safer sleep education, as infant care practices are likely rooted in strong cultural beliefs tied to infant well-being. The majority of caregivers endorsed that they would use a safe sleep intervention such as a baby box, which is an area that can be explored in future studies to see if this is a culturally acceptable and low-cost safe sleep solution for the community.

These study findings suggest the need to provide safer sleep education resources to healthcare professionals, community health workers and caregivers in this setting, particularly on topics such as bedsharing, minimizing objects in the sleep environment, sleep surface and cigarette smoke exposure, especially since these are identified to be protective against SIDS in high resource countries (2). A qualitative study of socio-cultural perceptions of SIDS among migrant Indian mothers residing in Sydney, Australia revealed that health messages aimed at lowering SIDS may be inappropriate for certain cultural groups for which the advice (e.g., putting a baby to sleep in a cot next to the parents' bed) may differ from the parents' cultural values and practices (e.g., the tradition of bedsharing) (17). It is thus important to recognize the role that cultural values and practices play, along with the socioeconomic, educational and health literacy disparities that may exist in these communities, and work closely with families, community health workers, health professionals and public health experts to cultivate messaging and solutions that are practical and culturally acceptable.

While this study was designed to be exploratory, the results were limited by a relatively low sample size. In our designated survey areas, few participants met the inclusion criteria of being a caregiver for an infant. Another study limitation was a potential reporting bias due to face-to-face interview methodology, as caregivers may have been reluctant to report substance use secondary to cultural stigma. Additionally, the convenience sampling approach may have resulted in selection bias. Other limitations includes data limitations due to a reliance on caregiver self-report rather than direct observation of sleep environment and interpretation gaps, as the study did not quantify relative risk or odds.

Future studies should focus on expanding the sample size and geographic locations of study, potentially through collaboration with government-run clinics/vaccine clinics. The topic of smoke exposure, pacifier use and sleep surface should also be further explored and characterized. While an anonymous survey would reduce interview bias, this method may not be feasible due to community literacy rates.

This study revealed that infant caregivers in Kolkata, India frequently described risk factors and protective factors that have been associated with SIDS in high resource communities. Caregivers reported rarely receiving guidance on infant sleep practices from health care providers. These are important findings that can inform future research and potential interventions (e.g., “baby box”) that are sustainable, socially and culturally appropriate and driven to promote safe infant sleep practices.

## Data Availability

The original contributions presented in the study are included in the article/Supplementary Material, further inquiries can be directed to the corresponding author.
