# Supplementary material for: Risk and protective factors for sudden infant death syndrome (SIDS) in low-resource communities in Kolkata India: a mixed methods exploratory study of semi-structured interviews and survey data
Source: Front Pediatr. 2025 Nov 20;13:1652669. doi: 10.3389/fped.2025.1652669 (PMC12675373; doi:10.3389/fped.2025.1652669)
Supplement: Supplementary file 3 [file Datasheet3.pdf]

**Semi-structured Group Interview # 1**

“How did you put your baby to sleep, when they were a newborn”
-in the back position
-after 5 months, they would turn to the left or right side

“Where did you learn that information?”
-hospital nurse at Dabli (?) hospital told them to sleep in back position, at time of delivery before discharge home
-doctors and nurses told them

“Why did they say for baby to sleep on back?”
[to prevent] vomiting through the nose, to prevent breathing problems

“Tell me of any challenges you faced when you came home with the baby”
-baby had a cough and cold after 15 days
-cold/cough fever after baby came home 5 days
-digestive problems, someone gave Zofran at 1.5 months

“Did you prepare a sleep space for baby when they came home”
-after (majority)

“What kind of bed does the baby sleep on, what sleeping space for baby when first came home”
-pillow on backside
-special bed (blue bed, photo), mom’s mother recommended it, Mom gave it to her when baby was 11-12 days of life, used it for 1 month
-during day sleep in hammock then take hammock down and baby sleeps inside at night
-small mattress on bottom, then pillows under head and backside
-Don’t buy anything or prepare anything until baby is born

“What are your thoughts on the blue bed/carrier”
*--this mom liked the blue bed. Placed it on the back. No one else in group has one, but would like to use it but don’t have it. Think baby would sleep well if they had it. Everyone would like to use it, would keep the baby safe. No danger. Mom feels safe and she’s not worried.*

“What kinds of dangers are you worried about for your baby”
-mosquitos, insects, rats
-use a net and coil
-turn lights on so rats will not come, also use solar lamps

“Does anybody smoke cigarettes in or outside the house?”
-smokes outside
-chews Pan

-grandfather sometimes smokes inside and outside
-father smokes

“Any wooden cooking fires?”
-inside the room, use wooden fires and stove
-Wooden fire outside the home
-Gas stove inside the room

“Showing other example of sleeping environment [Baby Box]. What do you think about it?”
-Very good
-Wouldn’t worry about ants or rats, would be worry free
-Everybody would like it (husband, sister in law)
-Thinks baby would be safe in it, baby will be fine

“Would you like to use something like this?”
-Yes

“Anything they don’t like about the baby box?”
-No

One focus group Mom asked “Is it possible to put a net on it?”
-possibly

“During first year of life, have you heard of a baby passing away during sleep?”
-In her village, she heard of this happening but at time of delivery
-Heard of this happening where a baby had a breathing problem, blanket was over her face, baby was about 1 year

“How did community react?”
-Does not know how the community reacted
-otherwise no

**Semi-structured Group Interview # 2**

Ages of children:
1.5 years, 10 mos, 1.5 years, 3 years, 9 years

“When baby first came home from hospital, how did you put your baby to sleep at night?”
-back position

“Did anyone tell you that specific information, like your Mom or at the hospital?”
-No

“What type of sleeping surface?”

-Bed, floor (hard surface), blanket with plastic
-When come home from hospital, they give some plastic mat, so they use that. Some hospitals give it

“Any environmental concerns (mosquitos, animals, flooding)?”
-none, mosquitos (uses a machine, electric mosquito repellant), mosquito net and coil
-told from hospital not to use mosquito coil b/c can cause health problems with the child

“Where did you have your baby?”
-state general hospital, private hospital
-Norpara state general

“Did they tell you anything in regards to how to put baby to sleep when you left hospital”
-nobody taught them, they just ‘knew’
-mother taught about sleeping on back
-learned from mother that that is how you should baby to sleep
-mother in law taught me how to put baby to sleep
-*Why? b/c Mother in law said better to sleep on the back b/c if you sleep on the side, baby’s bones stick out and you have to massage it to get bone in the neck back in*

“Did Mom ever say why to put baby on back?”
-So he doesn’t fall, maternal experience
-Baby’s grandmother watched baby after the delivery

“Anybody smoke outside or inside the home?”
-no smoking inside house
-gas stove
-somebody smokes inside the room
-kerosene oil stove

“Did you breastfeed your baby”
-everybody does

“Any other challenges?”
-had 2 spontaneous miscarriages, no other problems
-no complications, but at delivery baby didn’t cry right away. Eventually went home with mom
-no problems
-when she would bathe the baby, he couldn’t breathe

“Explain and show baby box. What do you think?”
-they like it

“What do you like about it?”
-baby is going to be safe, won’t fall out, nothing can touch baby in there
-cats and other animals can’t touch him

-baby falls from cot a lot, so this way baby won't fall out, will be safe. If she gets up and leaves, baby can fall, this way baby won't fall
- baby won't fall
-safe, won't fall

"Is there anything you do not like about this? Any faults in this?"
-worried about if you cover it, baby won't breathe
-no faults, likes it the way it is
-thinks it is fine
-can you put a mosquito net on it?, if there is a mosquito net then the mosquitos won't bite
-mosquito net would be helpful

"During first year of life, have you heard of a baby passing away during sleep?"
-Yes, one of the mom's had her child at 1 month die during sleep, it was her second child (middle child)—*said her child that passed away breastfed. She doesn't want to remember it or share*
-Sister's child died while sleeping at 3 days old, mouth was in open position, and when they woke up he passed away. Baby was born at home, was full term.
-Heard of delivery and it was a stillbirth
-She has heard about someone drinking milk and came out of baby's nose and died at that time

**<u>Semi-structured Group Interview # 3</u>**
Ages of children:
1.5 months, 3 months, 1 yo and 6 yo

"How do you put your baby to sleep at night?"
-initially on back but baby would turn to left or right side, she would sleep on a cot on a mattress or on a floor, would use pillow guard
-sleep on a bed/mattress
-sleep on a thick blanket on a cot

"Did anyone teach you/tell you how to put your baby to sleep?"
-Mother
-Doctor taught her, didn't tell a reason but put her on her back-Medical college hospital-Sagar-Dutta Hospital
-neighbor
-would lay baby on back and turn to her side, reason b/c at the hospital the doctor told them if they lay the baby on the back, the baby might get scared or lose their energy or something like that, so turn baby to side so baby wouldn't get scared-at Norapara Hospital

"Did they give any books or materials on how to put baby to sleep?"
-has a book, will show afterwards

“Can you talk about any challenges you faced when you came home?”
-first time when she came home from hospital, there was no problem, but after 1 month, baby had cold so baby required oxygen/nebulizer at hospital, did not stay at hospital
-no problems/challenges

“Any worries about bugs/mosquitos/flooding?”
-yes, about rats. She uses a mosquito net. No coils
-Only rats, nothing else
-Cockroaches
-No challenges

“How old were babies when they were born? Were any of them early?”
-10 months
-over dates

“Anybody smoke cigarettes in/outside home?”
-baby’s Dad, outside home

“Anybody use cooking fire inside or outside the house?”
-Gas stove, outside the room
-Inside the room, kerosene oil stove

Show baby box, this is a cardboard box with a mattress inside, with a sleeping place for baby. What are your thoughts? What do you like or not like about it?
-no fear from anything
-baby would not fall
-baby would be safe
-if they had it, the baby would be safe and they would use it

Is this something you would use?
-if had a mosquito net, would like it more
-needs a mosquito net

Sensitive question about if ever heard about infant dying during sleep during first year of life?
-baby had a cough/cold, went to sleep, mattress was covered from mouth

How did the community react?
-Sad, shocked, very sad
-Mother was crying at that time
-neighbor (to house where incident happened) was scared

<u>Notable quotes from individual questionnaire</u>
*“If baby sleeps on back, shape of head will be good”*
*–Mother from Nivedita colony*

*“Sleep on back [to prevent] vomiting through the nose, to prevent breathing problems”*
*-said by nurse or doctor in hospital to a Mother in Ambedkar colony*

*“Place baby on right side. If put on back, baby will die”*
*-Mother in Ambedkar colony, describing what state hospital personnel told based on a card*

“*sleep on the back b/c if [baby] sleep on the side, baby’s bones stick out and you have to massage it to get bone in the neck back in”-Mother at Ambedkar Colony*

*“would lay baby on back and turn to her side, reason b/c at the hospital the doctor told them if they lay the baby on the back, the baby might get scared or lose their energy or something like that, so turn baby to side so baby wouldn’t get scared”*-Mother at Nivdita colony on advice given to her at Norapara Hospital
