# Supplementary material for: Risk and protective factors for sudden infant death syndrome (SIDS) in low-resource communities in Kolkata India: a mixed methods exploratory study of semi-structured interviews and survey data
Source: Front Pediatr. 2025 Nov 20;13:1652669. doi: 10.3389/fped.2025.1652669 (PMC12675373; doi:10.3389/fped.2025.1652669)
Supplement: Supplementary file 4 [file Image1.pdf]

Semi-structured Interview Script

Theme 1: Sleeping Practices

"How do you put your baby to sleep? /Tell me how you put your baby to sleep"

- Follow up Questions
  - How many children do you have and what ages?
  - Was baby pre-term?
  - Where does your baby sleep?
  - What kind of surface (floor, mattress, mat) does your baby sleep on?
  - Does your baby sleep with any other items (eg. Blankets)?
  - Did you prepare a sleeping area for the baby before he/she was born?
  - Do you (or others) sleep in the same bed or sleeping area as your baby?
  - In what position do you place your child to sleep, and why?
  - Who watches the baby while he/she is sleeping?
  - Is there anything you do to ensure your baby sleeps safely? Who taught you this?
  - What environmental concerns do you have (if any)?

Theme 2: Assessing Risk Factors for SIDS

"Tell me how easy or difficult it is to take care of the baby"

- Follow up Questions
  - Is this your first baby?
  - Do you breastfeed your baby?
  - What was the most challenging thing you faced when you came home with your baby?
  - Did your baby have any health issues when he/she was born?
  - Did you receive prenatal care? Where? Did they tell you anything about how baby should sleep during your prenatal visits and if so what did they tell you?

"Tell me about the home environment"

- Follow up Questions about the home
  - Does anyone smoke in or outside the house?
  - Does anyone use a cooking fire inside or outside the house and if so what type of fire?
  - How many people live in the home?
  - Where does everyone sleep?

Theme 3: Perception of SIDS/Unexpected Deaths during sleep (to ask parents as well as providers potentially)

"Have you ever heard of a baby unexpectedly dying during sleep during their first year of life?"

- If so, what did you think was the reason for the death?
- How did the community, parents of the baby react to this death?
- How many times have you heard of this happening?
