# Supplementary material for: Risk and protective factors for sudden infant death syndrome (SIDS) in low-resource communities in Kolkata India: a mixed methods exploratory study of semi-structured interviews and survey data
Source: Front Pediatr. 2025 Nov 20;13:1652669. doi: 10.3389/fped.2025.1652669 (PMC12675373; doi:10.3389/fped.2025.1652669)
Supplement: Supplementary file 5 [file Image2.pdf]

Infant Sleep Study Survey শিশুর ঘুম সম্বন্ধীয় সমীক্ষা

1. **What is your relationship to the baby?** আপনি সন্তানের সাথে কীভাবে সম্পর্কিত**?**
    a. **Mother** মা
    b. **Father** পিতা
    c. **Grandmother/Grandfather** ঠাকুমা / ঠাকুরদাদা
    d. **Aunt/Uncle** কাকী / কাকা
    e. **Friend/Neighbor** বন্ধু / প্রতিবেশী
    f. **Other** অন্যান্য

2. **How old is the youngest baby you care for?** আপনার যত্ন নেওয়া সবচেয়ে কম বয়সী শিশুর বয়স কত**?**
    a. **12 months or younger. 12** মাস বা তার চেয়ে কম বয়সী।
    b. **Over 12 months. Thank you for your participation in the survey. 12** মাসের বেশী বয়সী। সমীক্ষায় আপনার অংশগ্রহণের জন্য আপনাকে ধন্যবাদ।

3. **What is your baby's gender?** আপনার শিশুর লিঙ্গ কী**?**
    a. **Male** পুরুষ
    b. **Female** মহিলা

4. **Most of the time, are you the person that takes care of the baby?** আপনি কি সেই ব্যক্তি যিনি বেশিরভাগ সময় শিশুর যত্ন নেন**?**
    a. **Yes** হ্যাঁ
    b. **No. Thank you for participation in the survey** না। সমীক্ষায় অংশ নেওয়ার জন্য আপনাকে ধন্যবাদ

5. **What colony do you live in?** আপনি কোন কলোনীতে বাস করেন**?**
    a. **Nivedita Colony**
    b. **Ramkrishna Colony**
    c. **Dr. Ambedkar Colony**
    d. **New Metro Colony**
    e. **Any other**

6. **Where were you born?** আপনি কোথায় জন্মেছিলেন?
    a. **India** ভারতে
    b. **Other: ________** অন্যদেশেঃ________

7. **Does the baby's mother work outside the home?** শিশুর মা কি বাড়ির বাইরে কাজ করেন**?**
    a. **Yes** হ্যাঁ
    b. **No** না
    c. **Does not apply** এখানে প্রযোজ্য নয়

8. **Does the baby's father work outside the home?** সন্তানের বাবা কি বাড়ির বাইরে কাজ করেন**?**
    a. **Yes** হ্যাঁ
    b. **No** না
    c. **Does not apply** এখানে প্রযোজ্য নয়

9. **Does anyone else help take care of the baby since he/she was born?** জন্মের পর থেকে অন্য কেউ কি সন্তানের যত্ন নিতে সহায়তা করে**?**
    a. **Yes** হ্যাঁ
    b. **No → Go to question 11** না → প্রশ্ন **11** এ যান

10. **Who has helped care for the baby? (Choose all that apply)** বাচ্চার যত্নে কে সাহায্য করেছে**?** **(**একাধিক উত্তরও দিতে পারেন**)**
    a. **Baby's father** শিশুর বাবা
    b. **Baby's mother** শিশুর মা
    c. **Baby's grandmother/grandfather** শিশুর ঠাকুমা / দাদু
    d. **Baby's aunt/uncle** শিশুর কাকি / কাকা
    e. **Baby's brother/sister** শিশুর ভাই / বোন
    f. **Your friend/ Neighbor** আপনার বন্ধুরা/ প্রতিবেশী
    g. **Babysitter / Nanny** দ্য বেবিসিটার / আয়া
    h. **Other. Please tell us:________** অন্যান্য। দযাকরে আমাদের বলুন:**________**

11. **What was the age of the baby's birth mother during the delivery?** সন্তানের জন্মের সময় শিশুর মার বয়স কত ছিল**?**
    a. **0-19 years** 0-19 বছর
    b. **20-29 years** 20-29 বছর
    c. **30-39 years** 30-39 বছর
    d. **40-49 years** 40-49 বছর
    e. **50+ years** 50+ বছর

12. **What is your educational status?** আপনার শিক্ষাগত যোগ্যতা কি**?**
    a. **Primary education or less (1st-4th standard)** প্রাথমিক শিক্ষা (প্রথম শ্রেণী থেকে চতুর্থ শ্রেণী) বা তার চেয়ে কম
    b. **Secondary education (5th-10th standard)** মাধ্যমিক শিক্ষা (পঞ্চম শ্রেণী থেকে দশম শ্রেণী)
    c. **Higher secondary (11th-12th standard)** উচ্চ মাধ্যমিক (একাদশ-দ্বাদশ শ্রেণি)
    d. **College/Graduate School** কলেজ / স্নাতক শ্রেণি
    e. **Other education (for example technical school, trade school)** অন্যান্য শিক্ষা (উদাহরণস্বরূপ কারিগরী শিক্ষার স্কুল, ট্রেড স্কুল)

13. **How many other children do you currently care for?** আপনি বর্তমানে আরও কত বাচ্চাদের যত্ন নিচ্ছেন**?**
    a. **I don't care for any other children** আমি অন্য কোনও বাচ্চার যত্ন নিই না
    b. **Between 1 and 2 other children 1**টি বা **2**টি অন্যান্য শিশুদের
    c. **More than 2 other children** আরও **2** টির বেশী অন্য বাচ্চা

14. **How was the baby born?** শিশুটির জন্ম কীভাবে হয়েছিল**?**
    a. **Natural Delivery** সাধারন প্রসব
    b. **Planned C-section** সি**-**বিভাগ পরিকল্পনা করা হয়েছে
    c. **Emergency C-section** জরুরী সি**-**বিভাগ

15. **Where was the baby born?** বাচ্চাটি কোথায় জন্মগ্রহণ করেছিল**?**
    a. **At home** ঘরে
    b. **At the hospital** হাসপাতালে
    c. **At a clinic** একটি ক্লিনিকে
    d. **Other. Please tell us:** অন্যান্য। দযাকরে আমাদের বলুন:

16. **How many months was the baby when he or she was born?** বাচ্চা তার জন্মের আগে কত মাস মায়ের গর্ভে ছিল?
    a. **<7 months** সাত মাসের কম
    b. **7 months** সাত মাস
    c. **8 months** আট মাস
    d. **>=9 months** নয় মাস বা তার বেশী
    e. **Unsure ঠিক বলতে পারছি না**

17. **After the baby was born, was there any medical problems or suffering that required him or her to stay in the NICU?** সন্তানের জন্মের পরে, শিশু কি এতটা অসুস্থ ছিল যে তার একটি নিবিড় পরিচর্যা ইউনিট (এনআইসিইউ) এর অতিরিক্ত চিকিৎসার প্রয়োজন ছিল?
    a. **Yes** হ্যাঁ
    b. **No** না
    c. **Unsure** ঠিক বলতে পারছি না

The next questions are about where and how the baby sleeps. পরবর্তী প্রশ্নগুলি শিশু কোথায় এবং কীভাবে ঘুমায় সে সম্পর্কে।

18. Did anyone (ex.family or nurse or doctor) talk to you about how to lay the baby down to sleep? কীভাবে শিশুটিকে ঘুমাতে শুইয়ে রাখবেন (পরিবারের কেউ বা ডাক্তার) সে ব্যাপারে কি আপনার সাথে কথা বলেছেন?

a. **Yes** হ্যাঁ
b. **No → Go to question 22** না → প্রশ্ন 22 এ যান

19. Who talked with you about how to lay the baby down to sleep? (Choose all that apply) কীভাবে বাচ্চাকে শোয়াতে হয় ঘুমানোর জন্য হয় সে সম্পর্কে আপনার সাথে কে কথা বলেছেন? (একাধিক উত্তরও দিতে পারেন)

a. **OB/GYN doctor or nurse during the pregnancy check-ups** গর্ভাবস্থায় চেক-আপ করার সময় ওবি / জিওয়াইএন ডাক্তার বা নার্স
b. **Doctor or nurse in the nursery** নার্সারিতে ডাক্তার বা নার্স
c. **Baby's doctor** শিশুর ডাক্তার
d. **Family member** পরিবারের সদস্য
e. **Friend/Neighbor** বন্ধু / প্রতিবেশী
f. **Knowledge gathered from my own experience** আমার নিজের অভিজ্ঞতা থেকে জেনেছি
g. **Knowledge gained from electronic media (Internet, TV), books or magazines** বৈদ্যুতিন মাধ্যম (ইন্টারনেট, টিভি), বই বা ম্যাগাজিন থেকে জেনেছি
h. **Nobody** কেউ না
i. **Other. Please tell us:** অন্যান্য। দয়াকরে আমাদের বলুন:

20. Which position was recommended by the health care provider to lay the baby down to sleep? (Choose all that apply) স্বাস্থ্যসেবা সরবরাহকারী কোন অবস্থানে শিশুকে ঘুমোনোর জন্য শোয়ানোর পরামর্শ দিয়েছিলেন? (একাধিক উত্তরও দিতে পারেন)

a. **On his or her side** পাশ ফিরে
b. **On his or her back** চিৎ হয়ে
c. **On his or her stomach** উপুড় হয়ে
d. **Leaning on an object (such as pillow or blanket)** একটু উঁচু কিছুতে যেমন বালিশ বা কম্বলে হেলান দিয়ে

e. **No recommendations were given by a healthcare provider** স্বাস্থ্যসেবা প্রদানকারী কর্তৃক কোনও সুপারিশ করা হয়নি

21. Which position was recommended by family or friends to lay the baby down to sleep? (Choose all that apply) পরিবার বা বন্ধুরা কোন অবস্থানে শিশুকে ঘুমোনোর জন্য শোয়ানোর পরামর্শ দিয়েছিলেন? (একাধিক উত্তরও দিতে পারেন)

a. **On his or her side** পাশ ফিরে
b. **On his or her back** চিৎ হয়ে
c. **On his or her stomach** উপুড় হয়ে
d. **Leaning on an object (such as pillow or blanket)** একটু উঁচু কিছুতে যেমন বালিশ বা কম্বলে হেলান দিয়ে
e. **No recommendations were given by family or friends** পরিবার বা বন্ধুদের দ্বারা কোনও সুপারিশ করা হয়নি

22. Most of the time, how do you put your baby to sleep? (Choose all that apply) আপনি **বেশীরভাগ সময়ে** কোন অবস্থানে শিশুকে শুইয়ে রাখেন ঘুমানোর জন্য? (একাধিক উত্তরও দিতে পারেন)

a. **On his or her side** পাশ ফিরে
b. **On his or her back** চিৎ হয়ে
c. **On his or her stomach** উপুড় হয়ে
d. **Leaning on an object (such as pillow or blanket)** একটু উঁচু কিছুতে যেমন বালিশ বা কম্বলে হেলান দিয়ে

23. Why do you like this position? (Choose all that apply) আপনি কেন এই অবস্থানে বাচ্চাকে শোয়ানো পছন্দ করছেন? (একাধিক উত্তরও দিতে পারেন)

a. **Advice from family/friends** পরিবার / বন্ধুদের পরামর্শ
b. **Advice from health care provider** স্বাস্থ্যসেবা সরবরাহকারীর পরামর্শে
c. **Other advice** অন্যান্য পরামর্শ
d. **Knowledge gathered from my own experience** আমার নিজের অভিজ্ঞতা থেকে জেনেছি
e. **Knowledge gained from electronic media (Internet, TV), books or magazines** বৈদ্যুতিন মাধ্যম (ইন্টারনেট, টিভি), বই বা ম্যাগাজিন থেকে জেনেছি
f. **Baby's comfort** শিশুর আরাম হয় তাই
g. **Safety concerns** শিশুর সুরক্ষার জন্য
   Please list which safety concerns:_____________________ দয়া করে সুরক্ষার সাথে সম্পর্কিত বিষয়গুলির তালিকা দিন: _____________________
h. **I don't know** আমি জানি না
i. **Other. Please tell us:** অন্যান্য। দযাকরে আমাদের বলুন: ___________________

24. Please view images: where does the baby sleep during the day? (Choose all that apply) দয়া করে এই ছবিগুলো দেখুন: দিনের বেলা শিশুটি কোথায় ঘুমায়? (একাধিক উত্তরও দিতে পারেন)

a. **Crib** ডাবা
b. **Baby's Bed (Portable bed)** শিশুদের বিছানা (সহজে বহন করা যায় এমন)
c. **Hammock** হ্যামক
d. **Bassinet** ঢাক্নিযুক্ত বেতের দোলনা
e. **Car seat** গাড়ির সিট
f. **Playpen** প্লে-পেন
g. **Parent's bed Soft Mattress** পিতামাতার নরম বিছানা
h. **Parent's bed Hard Surface (e.g. Cot, Chowki)** পিতামাতার শক্ত বিছানা (যেমন খাট, চৌকি)
i. **Rocker** আন্দোলক
j. **Mosquito net bed** মশারীযুক্ত শিশুদের বিছানা

k. **Floor** মেঝে
l. **Other. Please tell us:** অন্যান্য। দযাকরে আমাদের বলুন: ____________________

25. Where does the baby sleep during the night? (Choose all that apply) 26. রাতের বেলা শিশুটি কোথায় ঘুমায়? (একাধিক উত্তরও দিতে পারেন)

a. **Crib** ডাবা
b. **Baby's Bed (Portable bed)** শিশুদের বিছানা (সহজে বহন করা যায় এমন)
c. **Hammock** হ্যামক
d. **Bassinet** ঢাক্নিযুক্ত বেতের দোলনা
e. **Car seat** গাড়ির সিট
f. **Playpen** প্লে-পেন
g. **Parent's bed Soft Mattress** পিতামাতার নরম বিছানা
h. **Parent's bed Hard Surface (e.g. Cot, Chowki)** বাবা-মার শক্ত বিছানা (যেমন খাট, চৌকি)
i. **Rocker** আন্দোলক
j. **Mosquito net bed** মশারীযুক্ত শিশুদের বিছানা
k. **Floor** মেঝে
l. **Other. Please tell us:** অন্যান্য। দযাকরে আমাদের বলুন: ____________________

26. Some mothers in certain countries lay their babies to sleep in a box-bed like the one below. Is this something you would consider putting your baby to sleep in? কিছু দেশের মায়েদের নীচের মত বাক্স বেডে ঘুমানোর জন্য বাচ্চাদের শুয়ে রাখেন। আপনি কি আপনার বাচ্চাকে ঘুমিয়ে রাখার বিষয়টি বিবেচনা করবেন?

a. **Yes → Go to question 28** হ্যাঁ → প্রশ্ন 28 এ যান
b. **No** না

27. Please tell us why you would not use a box-bed like the one above. (Show picture) (Choose all that apply) দয়া করে আমাদের বলুন যে আপনি উপরের মতো বাক্স-বিছানা কেন ব্যবহার করবেন না। (ছবি দেখান) (একাধিক উত্তরও দিতে পারেন)

a. **I don't like the way it looks** আমি দেখতে দেখতে পছন্দ করি না
b. **I don't want to put my baby in a box** আমি আমার বাচ্চাকে একটি বাক্সে রাখতে চাই না
c. **I don't want to put my baby on the floor** আমি আমার বাচ্চাকে মেঝেতে রাখতে চাই না
d. **I don't think it is safe for my baby** আমি মনে করি না এটি আমার সন্তানের পক্ষে নিরাপদ
e. **I prefer to have my baby in my bed** আমি আমার বিছানায় আমার শিশুকে পছন্দ করি
f. **Other. Please tell us:** অন্যান্য। দযাকরে আমাদের বলুন:__________________________

28. Which of the following items are in bed with the baby? (Choose all that apply) নীচের কোন আইটেমটি শিশুর সাথে বিছানায় রয়েছে? (একাধিক উত্তরও দিতে পারেন)

a. **Pillow** বালিশ
b. **Blanket** কম্বল
c. **Soft mattress** নরম গদি
d. **Soft toys like stuffed animal** স্টাফড পশুর মতো নরম খেলনা
e. **Hard toys like rattles or toy cars** দড়ি বা খেলনা গাড়ির মতো শক্ত খেলনা
f. **Sheet** চাদর
g. **Clothes** বস্ত্র
h. **Diapers or Pampers** ডায়াপার বা প্যামার
i. **Bottle** বোতল
j. **Mosquito net** মশারি

k. **Other. Please tell us:** অন্যান্য। দযাকরে আমাদের বলুন:________________________

29. Does anybody else sleep in the area where the baby is sleeping? যেখানে শিশুটি ঘুমায় সেখানে কি আরো অন্য কেউ ঘুমায়?

a. **Yes** হ্যাঁ
b. **No** না

30. Does the baby use a pacifier when going to sleep? (see picture) ঘুমাতে যাওয়ার সময় শিশুটি কী প্রশান্তকারী ব্যবহার করে (ছবি দেখুন)

a. **Yes** হ্যাঁ
b. **No** না
c. **Sometimes** কখনও কখনও

31. Which of the following are you worried about being a danger to the baby in their home? (Choose all that apply) নীচের কোনটি বিষয়টি বাড়িতে শিশুদের জন্য বিপদজ্জনক হওয়ার বিষয়ে আপনি উদ্বিগ্ন? **(**একাধিক উত্তরও দিতে পারেন**)**

a. **Mosquitoes or other flying insects** মশা বা অন্যান্য উড়ন্ত পোকামাকড়
b. **Scorpions or other crawling insects** বিছে বা অন্যান্য সরীসৃপ জাতীয় পোকামাকড়
c. **Rats or other rodents** ইঁদুর বা অন্যান্য ইঁদুর জাতীয় কিছু
d. **Snakes** সাপ
e. **Flooding** বন্যা
f. **Other. Please tell us:** অন্যান্য। দযাকরে আমাদের বলুন:________________________

The next questions are about you baby's feeding. পরবর্তী প্রশ্নগুলি আপনার শিশুর খাওয়ানো সম্পর্কে।

32. Did the baby ever receive breast milk? বাচ্চা কি কখনও মায়ের দুধ পেয়েছিল**?**

a. **Yes → Go to question 34** হ্যাঁ →34 নং প্রশ্ন দেখুন
b. **No** না

33. What were the reasons for the baby not receiving breast milk. (Choose all that apply) শিশু বুকের দুধ না পাওয়ার কারণগুলি কী ছিল**? (**একাধিক উত্তরও দিতে পারেন**)**

a. **Mother was sick or on medicine** মা অসুস্থ ছিলেন বা ওষুধে ছিলেন
b. **There were other children to take care of** যত্ন নেওয়ার জন্য অন্যান্য শিশুও ছিল
c. **Mother had too many household duties** মায়ের অনেক বেশি বাড়ির দায়িত্ব ছিল
d. **Mother preferred to not give breast milk** মা বুকের দুধ না দেওয়া পছন্দ করেন
e. **Giving breast milk was too hard** বুকের দুধ দেওয়া খুব কঠিন ছিল
f. **Mother was advised not to give breast milk. Please tell us who advised mother not to give breast milk:________** মাকে বুকের দুধ না দেওয়ার পরামর্শ দেওয়া হয়েছিল। দয়া করে আমাদের বলুন কে মাকে বুকের দুধ না দেওয়ার পরামর্শ দিয়েছেন**: ________**
g. **I don't know** আমি জানি না
h. **Other. Please tell us:** অন্যান্য। দযাকরে আমাদের বলুন:__________________________

34. Is the baby currently drinking breast milk? শিশুটি বর্তমানে বুকের দুধ পান করছে**?**

a. **Yes → Go to Question 37** হ্যাঁ → প্রশ্ন 37 এ যান
b. **No** না

35. How long did the baby drink breast milk? শিশু কতক্ষণ মায়ের দুধ পান করে**?**

a. **Less than 2 weeks** 2 সপ্তাহেরও কম

b. **2 weeks to 2 months 2** সপ্তাহ থেকে **2** মাস
c. **Between 2 and 6 months** 2 থেকে 6 মাসের মধ্যে
d. **6-12 months 6-12** মাস

36. What were the reasons that the baby stopped receiving breast milk? (Choose all that apply) শিশুর বুকের দুধ নেওয়া বন্ধ করার কারণগুলি কী কী ছিল**?** (একাধিক উত্তরও দিতে পারেন)

a. **Difficulty latching or nursing** অসুস্থতা কাটা বা নার্সিং
b. **Breast milk alone did not satisfy hunger** একা মায়ের দুধ খিদে মেটেনি
c. **Baby was not gaining enough weight** শিশুর যথেষ্ট পরিমাণ ওজন বাড়ছিল না
d. **Mother's nipples were sore, cracked, or bleeding** মায়ের স্তনবৃন্তগুলি কালশিটে, ফাটা বা রক্তক্ষরণে ছিল
e. **Giving breast milk was too hard, painful, or too time consuming** বুকের দুধ দেওয়া খুব কঠিন, বেদনাদায়ক বা খুব বেশি সময়সাপেক্ষ ছিল
f. **Mother was not producing enough milk, or her milk dried up** মা পর্যাপ্ত দুধ উত্পাদন করছিলেন না, বা তার দুধ শুকিয়ে গেল
g. **Too many other household duties** পরিবারের অন্যান্য অনেক দায়িত্ব
h. **It was the right time to stop breastfeeding** স্তন্যপান করানো বন্ধ করার সঠিক সময় ছিল
i. **Mother became sick or had to stop for other medical reasons** মা অসুস্থ হয়ে পড়েছিলেন বা অন্যান্য চিকিত্সার কারণে তাকে থামাতে হয়েছিল
j. **Mother went back to work or school** মা আবার কাজে বা স্কুলে ফিরে গেলেন
k. **Baby was jaundiced (yellowing of the skin or whites of the eyes)** শিশুর জন্ডিস হয়েছে (ত্বকের হলুদ হওয়া বা চোখের সাদা অংশ)
l. **Mother was advised to stop giving the breast milk. Please tell us who recommended that the baby stop receiving breast milk and tell us why:___________** মাকে বুকের দুধ দেওয়া বন্ধ করার পরামর্শ দেওয়া হয়েছিল। কারা বাচ্চাকে বুকের দুধ খাওয়ানো বন্ধ করার পরামর্শ দিয়েছিলেন তা আমাদের বলুন দয়া করে বলুন: ___________
m. **Other. Please tell us:** অন্যান্য। দযাকরে আমাদের বলুন:_____________________________

The next questions are about your baby's healthcare. পরবর্তী প্রশ্নগুলি আপনার শিশুর স্বাস্থ্যসেবা সম্পর্কে।

37. Did you take your baby for all of their vaccinations? আপনি কি আপনার বাচ্চাকে সমস্ত টিকা দিইয়েছেন?

a. **Yes, all of them** হ্যাঁ, এগুলি সব
b. **Yes, most of them** হ্যাঁ, তাদের বেশিরভাগই
c. **Yes, some of them** হ্যাঁ, তাদের মধ্যে কিছু
d. **No, baby is less than 2 weeks old** না, বাচ্চা 2 সপ্তাহেরও কম বয়সী
e. **No** না
f. **Unsure** ঠিক বলতে পারছি না

38. Do you certainly take your baby for routine checkups? আপনি কি অবশ্যই বাচ্চাকে নিয়মিত স্বাস্থ্য পরীক্ষার জন্য নিয়ে যান?

a. **Yes, all of them → Go to Question 40** হ্যাঁ, এগুলি সব → প্রশ্ন 40 তে যান
b. **Yes, most of them** হ্যাঁ, তাদের বেশিরভাগই
c. **Yes, some of them** হ্যাঁ, তাদের মধ্যে কিছু
d. **No, baby is less than 2 weeks old** না, বাচ্চা 2 সপ্তাহেরও কম বয়সী
e. **Only when sick** শুধু যখন অসুস্থ হয় কেবল তখনই
f. **No** না
g. **Unsure** ঠিক বলতে পারছি না

39. What was the reason at that time you did not go to all check-ups? (Check all that apply) নিম্নলিখিত কোনও কারণে শিশুটিকে রুটিন মেডিকেল চেক আপগুলি হয় নি? (সকল আবেদন যাচাই কর)

a. **Appointment times were inconvenient** অ্যাপয়েন্টমেন্ট সময় অসুবিধাজনক ছিল
b. **Did not have enough money to pay to visits** ভিজিটে টাকা দেওয়ার মতো পরিমাণ টাকা ছিল না
c. **Did not have transportation to get to the clinic or doctor's office** ক্লিনিক বা ডাক্তারের অফিসে যাওয়ার জন্য পরিবহন ছিল না
d. **There were too many other responsibilities** আরও অনেক দায়িত্ব ছিল
e. **There were other children to take care of** যত্ন নেওয়ার জন্য অন্যান্য শিশুও ছিল
f. **Mother was unaware of regular check-ups** মা নিয়মিত চেক-আপ সম্পর্কে অসচেতন ছিলেন
g. **Mother does not feel child needs regular check-ups?** মা কি মনে করেন না বাচ্চার নিয়মিত চেক-আপ করা দরকার?
h. **Other. Please tell us:** অন্যান্য। দযাকরে আমাদের বলুন:______________________________

The next questions are about the prenatal care received during the baby's birth mother's pregnancy. Prenatal care is regular pregnancy checkups with a doctor or nurse before your baby was born to monitor the pregnancy. পরবর্তী প্রশ্নগুলি শিশুর জন্ম মায়ের গর্ভাবস্থায় প্রাপ্ত প্রসবপূর্ব যত্ন সম্পর্কে। প্রসবকালীন যত্ন হ'ল গর্ভাবস্থা নিরীক্ষণের জন্য আপনার সন্তানের জন্মের আগে নিয়মিত গর্ভাবস্থায় চিকিত্সক বা নার্সের সাথে চেকআপ করা।

40. Did the baby's birth mother get regular pregnancy check-ups? শিশুর মা কি নিয়মিত গর্ভাবস্থার চেক আপ করান?

a. **Yes→ Go to Question 42** হ্যাঁ → প্রশ্ন **42** এ যান
b. **No** না
c. **Unsure → Go to Question 42** অনিশ্চিত → প্রশ্ন **42** এ যান

41. Were any of the following reasons an obstacle to getting pregnancy check-ups? (Choose all that apply) নিম্নলিখিত কোনও কারণে কি গর্ভাবস্থা চেক-আপ করাতে বাধা ছিল? (একাধিক উত্তরও দিতে পারেন)
If you did not get pregnancy check-ups, go to Question 43 যদি আপনি গর্ভাবস্থা চেক আপ না পান তবে প্রশ্ন **43** এ যান

a. **Could not get an appointment when convenient** সুবিধাজনক অ্যাপয়েন্টমেন্ট পাওয়া যায়নি
b. **Did not have enough money to pay for visits** ভিজিটের জন্য দেওয়ার মতো টাকা ছিল না
c. **Did not have transportation to get to the clinic or doctor's office** ক্লিনিক বা ডাক্তারের অফিসে যাওয়ার জন্য পরিবহন ছিল না
d. **Too many other things going on** আরও অনেক কিছুই অসুবিধা ছিল
e. **Could not take time off from work or school** কাজ বা স্কুল থেকে ছুটি নিতে পারেনি
f. **Did not have anyone to take care of other children** অন্য বাচ্চাদের দেখার নেওয়ার মতো কেউ ছিল না
g. **Was not aware of pregnancy** গর্ভাবস্থা সম্পর্কে সচেতনতা ছিল না
h. **Did not want anyone else to know about pregnancy** গর্ভাবস্থার বিষয়ে অন্য কাউকে জানাতে চাননি
i. **Did not want pregnancy check-ups** গর্ভাবস্থা চেক আপ চান না
j. **Unsure** ঠিক বলতে পারছি না

42. During your pregnancy, did any doctor, nurse or other health care worker talk to you about any of the following precautions or topics? (Choose all that apply) Please count only discussions, not reading materials or videos. আপনার গর্ভাবস্থায়, কোনও ডাক্তার, নার্স বা অন্যান্য স্বাস্থ্যকর্মী

নীচের কোন সতর্কতা বা বিষয়গুলির ব্যাপারে আপনার সাথে কথা বলেছেন? দয়া করে শুধু আলোচনাগুলিই বিবেচনা করবেন, লিখিত সামগ্রী বা ভিডিও নয়। (একাধিক উত্তরও দিতে পারেন)

a. **How to put the baby to sleep** কীভাবে বাচ্চাকে শূইয়ে ঘুম পাড়াতে হবে
b. **How smoking during pregnancy could affect baby** গর্ভাবস্থায় ধূমপান কীভাবে শিশুর ক্ষতি করতে পারে
c. **Breastfeeding baby** শিশুকে বুকের দুধ খাওয়ানোর নিয়ম
d. **How drinking alcohol during pregnancy could affect the baby** গর্ভাবস্থায় মদ্য পান কীভাবে শিশুর ক্ষতি করতে পারে
e. **How using narcotic drugs could affect the baby** কীভাবে মাদক ওষুধের ব্যবহার শিশুর উপর প্রভাব ফেলতে পারে
f. **How antibiotics or other medications could affect baby** অ্যান্টিবায়োটিক বা অন্যান্য ওষুধগুলি কীভাবে শিশুকে প্রভাবিত করতে পারে
g. **Unsure** ঠিক বলতে পারছি না

The next questions are about caregiver's use of alcohol and tobacco. You do not have to answer the question if you are not comfortable with. Please remember information here is confidential and voluntary. Your answers will only be used for research purposes. পরবর্তী প্রশ্নগুলি শিশুর যত্নকারীর মদ এবং তামাকের ব্যবহার সম্পর্কে। **আপনি যদি স্বাচ্ছন্দ্য বোধ না করেন তবে আপনাকে এই প্রশ্নের উত্তর দিতে হবে না।** দয়া করে মনে রাখবেন এখানে সব তথ্য গোপনীয় এবং স্বেচ্ছাপ্রদত্ত। আপনার উত্তরগুলি কেবল গবেষণার উদ্দেশ্যেই ব্যবহৃত হবে।

If you are not the Mother, please go to question 45 আপনি যদি মা না হন তবে অনুগ্রহ করে 45 নম্বর প্রশ্নে যান

43. Did you have any addictions (e.g. smoking, tobacco, alcohol, drugs) during your pregnancy or after? আপনার গর্ভাবস্থায় বা তার পরে আপনার কোনও আসক্তি (যেমন ধূমপান, তামাক সেবন, মদ্যপান, ড্রাগ) রয়েছে?

a. **Yes** হ্যাঁ
b. **No--> go to question 47** না -> প্রশ্ন 46 এ যান

44. What addictions did you have during pregnancy?(Choose all that apply) গর্ভাবস্থায় আপনার কোন নেশা ছিল? (একাধিক উত্তরও দিতে পারেন)

a. **Smoking** ধূমপান
b. **Chewing Tobacco** তামাক সেবন
c. **Alcohol** মদ
d. **Drugs** ড্রাগ
e. **Other:** অন্যান্য: ___________________________

45. What addictions (e.g. smoking, tobacco, alcohol, drugs) do you have currently? (Choose all that apply) আপনার বর্তমানে কি কোনো নেশা (যেমন ধূমপান, তামাক, মদ, ড্রাগ) রয়েছে? (একাধিক উত্তরও দিতে পারেন)

f. **Smoking** ধূমপান
g. **Chewing Tobacco** তামাক সেবন
h. **Alcohol** মদ
i. **Drugs** ড্রাগ
j. **Other:**________________ অন্যান্য: _________________

k. **None-->Go to question 47** না কিছুই নয় -> 47নং প্রশ্নে যান

46. Do you take any addictions when taking care of the baby? শিশুর যত্ন নেওয়ার সময় আপনি কি কোনও নেশা করেন?

a. **Yes হ্যাঁ**
b. **No না**

47. Does anybody in the household smoke cigarettes or cigars? পরিবারের কেউ কি ধূমপান করেন?

a. **Yes হ্যাঁ**
b. **No না**

48. What is the fuel source for cooking in the house?(Choose all that apply) ঘরে রান্নার জন্য জ্বালানীর উৎস কী (একাধিক উত্তরও দিতে পারেন)

a. **Wood / Coal burning** কাঠ / কয়লা জ্বালা
b. **Kerosene** কেরোসিন তেল জ্বালা
c. **Gas (e.g. LPG)** গ্যাস (উদাঃ এলপিজি)
d. **Other:___________** অন্যান্য: ____________

49. Do you think your baby gets exposed to any smoke? আপনি কি মনে করেন যে আপনার শিশু কোনও ধোঁয়ার সংস্পর্শে থাকে?

a. **Yes -->go to question 50** হ্যাঁ -> প্রশ্ন 50 এ যান
b. **No-->Thank you, end of survey** না -> জরিপ শেষে আপনাকে ধন্যবাদ

50. What kind of smoke is your baby exposed to? (Choose all that apply) আপনার শিশু কী ধরণের ধোঁয়ায় আক্রান্ত হয়েছে (একাধিক উত্তরও দিতে পারেন)

a. **Smoke from Cigarettes** সিগারেটের ধোঁয়া থেকে
b. **Smoke from wood burning for cooking** রান্নার জন্য কাঠ পোড়ানো থেকে ধোঁয়া
c. **Smoke from coal burning for cooking** রান্নার জন্য কয়লা পোড়ানো থেকে ধোঁয়া
d. **Smoke from Kerosene burning for Cooking** রান্নার জন্য কেরোসিনের আগুন থেকে ধোঁয়া
e. **Smoke from Mosquito repellent Coils burning** মশা তাড়ানোর কয়েল পোড়ানো থেকে ধোঁয়া
f. **Other:___________** অন্যান্য: ____________

Thank you for your participation in this survey. Your answers are very important to us and will help us understand how we can help make babies as safe as possible during sleep in Kolkata. এই সমীক্ষায় আপনার অংশগ্রহণের জন্য আপনাকে ধন্যবাদ। আপনার উত্তরগুলি আমাদের কাছে অত্যন্ত গুরুত্বপূর্ণ এবং কলকাতায় ঘুমের সময় আমরা কীভাবে শিশুদের যতটা সম্ভব সুরক্ষিত করতে সহায়তা করতে পারি তা বুঝতে আমাদের সহায়তা করবে।
